# Assessment of Suitable Habitats and Identification of Key Protection Areas for *Polyplectron katsumatae* in Jianfengling, Hainan Province, China

**DOI:** 10.3390/life15050826

**Published:** 2025-05-21

**Authors:** Wutao Yao, Yong Ma, An Long, Lixi Liu, Erping Shang, Shuyan Zhang, Jin Yang, Tianxiong Gao

**Affiliations:** 1 Key Laboratory of Earth Observation of Hainan Province, Hainan Aerospace Information Research Institute, Sanya 572029, China; yaowt@aircas.ac.cn (W.Y.); llxsdyp@bjfu.edu.cn (L.L.); shangep@aircas.ac.cn (E.S.); zhangshuyan@aircas.ac.cn (S.Z.); 2 Aerospace Information Research Institute, Chinese Academy of Sciences, Beijing 100094, China; yangjin@aircas.ac.cn (J.Y.); gaotianxiong23@mails.ucas.ac.cn (T.G.); 3 Environmental Emergency and Accident Investigation Center of Guangxi Zhuang Autonomous Region, Nanning 530028, China

**Keywords:** ESDM, Marxan model, ground-dwelling bird species, environmental preferences, evaluate the habitat suitability

## Abstract

*Polyplectron katsumatae* is a rare and endangered species endemic to Hainan, China. It has long been regarded as a subspecies of the widely distributed Grey Peacock-Pheasant (*Polyplectron bicalcaratum*), a classification that has resulted in a paucity of targeted conservation studies and rendered efforts to protect and restore its populations and habitats exceedingly challenging. In this study, the Jianfengling section of Hainan Tropical Rainforest National Park was designated as the research area. We comprehensively utilized infrared camera monitoring data for *P. katsumatae* and other species, alongside habitat environmental factor data obtained through multiple monitoring approaches. An ensemble species distribution model (ESDM) was employed to evaluate the habitat suitability for four ground-dwelling bird species, including *P. katsumatae*, and to investigate their environmental preferences and competitive interactions during habitat selection. Subsequently, the Marxan model was applied to identify key protection areas for *P. katsumatae*. The results indicate that the suitable habitat for *P. katsumatae* is primarily distributed in the central, eastern, and certain southern areas of the study region, with low spatial overlap and minimal competition from the suitable habitats of the other three ground-dwelling bird species. However, due to anthropogenic disturbances and the inherently stringent habitat requirements of *P. katsumatae*, its overall suitable habitat area is limited, exhibiting a concentrated distribution overall with fragmented, small patches within it. Our study recommends designating the eastern and southern regions of the study area as key protection areas for *P. katsumatae*, thereby providing a robust baseline environment and policy support for the targeted protection of its habitat and the recovery of its populations.

## 1. Introduction

Wildlife habitats are critical areas for sustaining biodiversity and ecosystem functions, and their quality significantly influences ecosystem stability [1]. Tropical rainforests, as one of the most biodiverse ecosystems in the world [2,3], serve as the primary habitat for numerous wild plant and animal species [4] and play an irreplaceable role in the conservation of genetic diversity and species preservation [5]. However, with the intensification of human activities and the ongoing impacts of climate change, global tropical rainforests face significant risks of fragmentation, degradation, and even loss [6,7], which severely affect the survival of many rare plant and animal species. To better protect the biodiversity of tropical rainforests and rare species, it is imperative to conduct comprehensive and scientific assessments of tropical rainforest habitats and to promote targeted species conservation by identifying key protection areas for different species.

Hainan’s tropical rainforests represent the primary distribution area of tropical rainforests in China [8], and due to their unique insular isolation, they serve as the sole habitat for many endemic animal species. Hainan Gibbon (*Nomascus hainanus*), Hainan Peacock-Pheasant (*Polyplectron katsumatae*), and Hainan Partridge (*Arborophila ardens*) are all rare and protected animal species endemic to Hainan. However, rapid socioeconomic development in Hainan has greatly impacted the habitats of wild flora and fauna in its tropical rainforests. For instance, *N. hainanus*, once widely distributed in Hainan, saw its population decline to as few as 13 individuals at its most critical period [9], nearing extinction in the wild. Thanks to targeted conservation efforts in recent years, the population has recovered to seven groups comprising 42 individuals as of February 2024. However, compared to well-known “flagship species” such as *N. hainanus*, many other endemic species in the region receive limited attention and fewer targeted conservation measures, leaving them in a critically endangered state. Notably, *P. katsumatae*, a rare and endangered species, had long been considered a subspecies of the widely distributed Grey Peacock-Pheasant (*Polyplectron bicalcaratum*) until it was classified as endangered by the IUCN in 2010 [10,11]; this has resulted in a paucity of targeted conservation research on *P. katsumatae*. The global population of *P. katsumatae* is inferred to range between 250 and 1000 individuals based on historical data and indirect observations, with significant uncertainty in these estimates. The current population is predominantly concentrated in the montane evergreen broadleaf forests spanning southwestern to central Hainan Island. The available limited studies indicate that habitat destruction, human hunting, and anthropogenic disturbances constitute the primary drivers of its endangered status [12,13,14], compounded by interspecific competition within its remaining suitable habitats. Currently, critical knowledge gaps persist regarding the species’ historical and contemporary distribution patterns, habitat preferences, and environmental requirements; even its current population size remains uncertain, further complicating targeted conservation efforts. Thus, initiating research on the suitable habitats of *P. katsumatae* is urgent.

Hainan tropical rainforests feature rugged terrain and lush vegetation. *P. katsumatae* is highly sensitive to human activities and adept at concealing its presence within dense foliage, making it extremely challenging to ascertain its population, distribution, and habitat status through field surveys. Currently, the use of infrared camera surveys to gather animal data [15], in conjunction with multi-source approaches such as satellite remote sensing, station monitoring, and field investigations to acquire habitat information [16,17], has become a key method in habitat assessment research. Meanwhile, species distribution models effectively integrate animal occurrence data with habitat environmental data, enabling the prediction of species distribution and the accurate evaluation of habitat suitability, and have thus become indispensable tools in research. This greatly facilitates research on the habitats of *P. katsumatae*. Currently, commonly used species distribution models include the Maximum Entropy Model (MaxEnt) [18], Random Forest Model (RF) [19], Support Vector Machines Model (SVM) [20], Generalized Linear Models (GLM) [21], and Generalized Additive Models (GAM) [22], among others. Different models offer distinct advantages and differ in their suitability for various data qualities and application scenarios. To enhance model applicability and robustness [23] and ensure the accuracy of habitat suitability predictions, ensemble species distribution model (ESDM) has been proposed and is widely applied in multi-species habitat assessment studies at regional scales. For example, Lawer [24] employed an ESDM to investigate the current distribution and influencing factors of suitable habitats for *Necrosyrtes monachus*, and to predict changes in its habitat suitability over the coming decades; similarly, Shivambu et al. [25] used an ESDM to evaluate the potential suitable habitats of various invasive bird species in South Africa, suggesting that densely populated urban landscapes are at high risk of pet bird invasions. Additionally, the use of the Marxan model provides a reliable method for effectively identifying key protection areas [26,27]. This model, based on evaluated habitat suitability, fully considers the connectivity and compactness of suitable habitats, along with conservation objectives and associated costs, to determine the optimal solution for key protection areas. For instance, Dehaghi et al. [28] employed multi-criteria evaluation and the Marxan model to delineate high-priority habitats for *Alectoris chukar* and *Phasianus colchicus* in Iran, while Wang et al. [29] utilized MaxEnt and Marxan to identify key protection areas for biodiversity in China’s southern Taihang region.

In this study, we comprehensively utilized infrared camera monitoring data and habitat environmental factor data obtained through multi-source monitoring methods in the Jianfengling research area of the Hainan Tropical Rainforest. Focusing on *P. katsumatae*—a rare and endangered species—and three other ground-dwelling bird species (*Lophura nycthemera*, *Garrulax maesi*, and *Garrulax pectoralis*) that are more widely distributed and may compete with *P. katsumatae*, we employed ESDM to analyze the spatial distribution of suitable habitats for these four species and the key factors influencing their distribution, and to investigate the interrelationships between species survival and habitat selection based on habitat overlap and species characteristics. Finally, the Marxan model was applied to identify key protection areas for *P. katsumatae*, thereby providing scientific recommendations for its targeted protection and that of its habitat. This study offers a theoretical basis and methodological guidance for formulating conservation strategies for rare species in Hainan Tropical Rainforest National Park.

## 2. Materials and Methods

### 2.1. Study Area

The Jianfengling sector (659.7 km^2^) constitutes a critical research zone within Hainan Tropical Rainforest National Park, geographically positioned in 108.73° E–109.03° E and 18.58° N–18.87° N. This biodiversity hotspot administratively straddles Dongfang County and Ledong City in southwestern Hainan Province. It is the lowest-latitude tropical rainforest in China, featuring the most complete vertical vegetation structure, the largest area, and the best preservation among the existing tropical rainforests in the country. Located at the tropical northern margin, Jianfengling exhibits distinct tropical monsoon climate features with pronounced seasonality. The region undergoes intensive convective precipitation coupled with high temperatures during summer months, creating persistently humid conditions, while winter months present markedly drier and cooler atmospheric conditions. The annual mean temperature is 24.5 °C, with extreme low temperatures reaching −2.8 °C and extreme high temperatures of 38.1 °C. The annual average rainfall is 2265.8 mm, with the highest annual rainfall reaching 3051.3 mm and the lowest 1470.1 mm, most of which falls between May and October [30]. Rainfall increases with elevation, with high-altitude areas receiving up to 3600 mm of precipitation. The topography of the study area is predominantly mountainous, with the highest peak reaching 1412 m. Influenced by climate, topography, and soil conditions, a vertical vegetation structure has developed from low to high altitudes, including tropical semi-deciduous seasonal rainforests, tropical evergreen seasonal rainforests, tropical northern-edge valley rainforests, tropical montane rainforests, tropical montane evergreen broadleaf forests, and montane moss-dominated dwarf forests [14]. The study area is rich in biodiversity, and long-term surveys have documented 2222 species of invertebrates and 400 species of vertebrates [31], including numerous rare and endangered species, such as *P. katsumatae*, *A. ardens*, *Manis pentadactyla*, *Macaca mulatta*, and *Prionailurus bengalensis*.

In recent years, tourism in Jianfengling has developed rapidly, and the research area includes several tourist attractions, which inevitably impose certain disturbances on the tropical rainforest ecosystem. Furthermore, numerous minority villages are located around the protected area, and some minority residents continue to practice traditional activities such as mountain travel and hunting. In addition, incursions by outsiders for resource extraction and even poaching have frequently occurred. Available studies [12,13,14] suggest that these activities could pose substantial risks to *P. katsumatae* populations and associated ecosystems, potentially leading to habitat degradation and biodiversity loss.

### 2.2. Species Distribution Data

**Figure 1:**
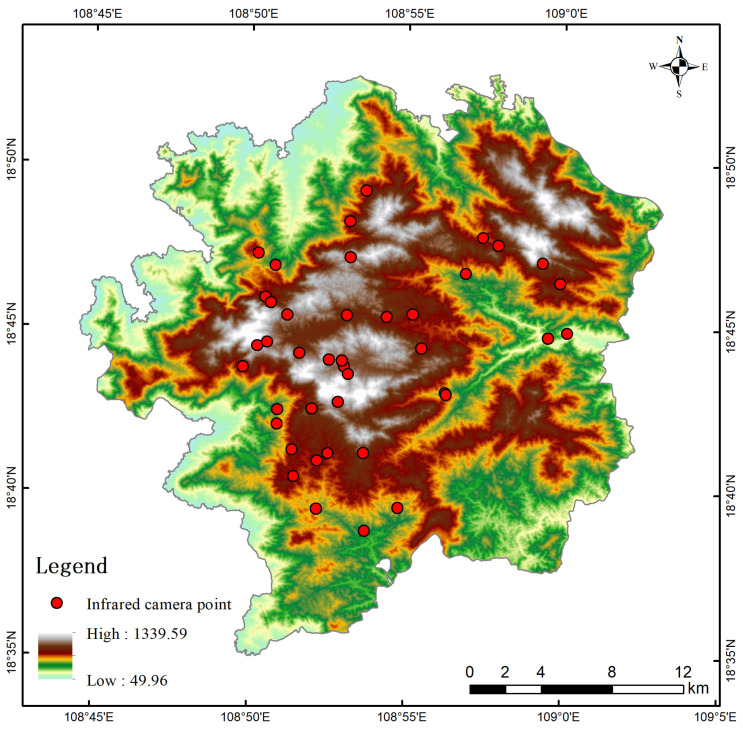
Elevation distribution and infrared camera monitoring points in the study area.

Between October 2013 and February 2019, the study area was equipped with 132 infrared cameras, installed in multiple phases [14] (Figure 1), to conduct a long-term survey of ground-dwelling birds and mammals. Due to the complex topography and dense rainforest vegetation in the study area, many regions are inaccessible to human researchers. Additionally, the lack of signal coverage in mountainous areas necessitates the manual retrieval of infrared camera data, resulting in significant challenges for wildlife data collection. Consequently, the existing camera trap deployment sites are not uniformly distributed across all zones of the study area. However, the existing camera sites are sufficiently numerous, exhibit high environmental heterogeneity across their distribution, and span an extended data collection period. These attributes collectively provide robust support for the study. Over a monitoring period of more than five years, a total of 42,563 camera days were recorded, yielding 19,953 identifiable images, including 18,467 of mammals and 1486 of birds. A total of 62 species of wild animals were accurately identified, belonging to 16 orders and 33 families. Among them, mammals comprised 16 species from 6 orders and 11 families, while birds included 46 species from 10 orders and 22 families. This study primarily focused on *P. katsumatae*. Additionally, based on survey data, three other ground-dwelling bird species—*L. nycthemera*, *G. maesi*, and *G. pectoralis*—were selected for comparative analysis due to their relatively high abundance and potential competition with *P. katsumatae*. The conservation status and key ecological characteristics of these four species are presented in Table 1 and Table 2.

### 2.3. Environmental Factor Data

The environmental factor data used in this study include 6 categories: climate data [35], water data, road data [36,37], land use/cover data [38], vegetation data [39], and topographic data [40]. These datasets were sourced from different origins, with some categories containing multiple subcategories of environmental factors (Table 3). All data underwent standardization processes and were prepared as analysis-ready data products. For analytical convenience, all datasets were resampled to a 30 m resolution. Multicollinear variables were eliminated based on two criteria: VIF exceeding 10 (calculated via R’s usdm package v4.1.1) and Pearson’s r ≥ 0.7 (analyzed using Python 3.9′s Seaborn) [41,42,43].

### 2.4. Species Distribution Modeling and Accuracy Evaluation

ESDM was employed to assess the habitat suitability of *P. katsumatae* and three other species. The ESDM integrates three widely used and effective models for habitat prediction—MaxEnt, SVM, and RF. The ensemble performance of the ESDM effectively ensures the precision and stability of evaluation results, partially mitigating the adverse effects caused by the uneven spatial distribution of infrared camera sites. All computational analyses were conducted in R 4.3.3 with the SSDM package (v0.2.9). Individual SDM predictions achieving AUC > 0.8 were selected for ensemble integration, where each model’s contribution was weighted proportionally to its AUC value relative to the total AUC of all included models. The calculation formula is as follows [31,44]:

(1) $$W_{i}=\frac{r_{i}}{\sum_{j=1}^{h}r_{j}}$$

In the above equation, $W_{i}$ represents the weight of the *i*-th model; $r_{i}$ denotes the AUC value of the *i*-th single model; and h represents the total number of single models with AUC values greater than 0.8.

All three single SDM analyses require species presence point data, which are derived from infrared camera survey results. In addition, the RF and SVM models also require absence data. These absence data were generated using the random selection method provided by the SSDM package. During model prediction, 80% of the species distribution data were randomly split as the training set, while the remaining data served as the test set. We employed 10-round bootstrap validation to ensure robustness.

The accuracy of the model predictions was evaluated using two performance metrics, AUC and TSS [45,46]. AUC values range from 0.5 to 1, with higher values indicating better predictive performance and lower values suggesting that the model’s performance is closer to random guessing. TSS spans from −1 to 1, where higher positive values (approaching 1) reflect better model accuracy, values around 0 suggest predictions no better than random, and negative values demonstrate poorer performance than random guessing. The final output of the ESDM is a raster dataset representing habitat suitability, with pixel values ranging from 0 to 1, and higher values denote better habitat suitability.

### 2.5. Identification of Key Protection Areas

In this study, the Marxan model [47] was employed, based on the ESDM prediction result, to identify the key protection areas for *P. katsumatae* within the Jianfengling research area. The Marxan model is grounded on the principles of Systematic Conservation Planning (SCP) and employs optimization algorithms to minimize conservation costs while meeting designated conservation objectives. The model’s calculation formula is as follows:

(2) $$\sum_{PU_{S}}Cost+BLM\sum_{PU_{S}}Boundary+\sum_{ConValue}SPF\times Penalty+CostThresholdPenalty(t)$$

where Cost is the sum of the costs in each planning unit to be protected; BLM (Border Length Modifier) is the border adjustment factor; SPF (Species Penalty Factor) is the penalty factor; Penalty is the penalty value, increased as necessary to meet the protection goal; and CostThresholdPenalty is a cost cap for the entire protection plan.

Because the Jianfengling research area is located within a national park, its conservation costs primarily involve limiting unnecessary anthropogenic disturbances during the protection of *P. katsumatae*. Therefore, in identifying the key protection areas for *P. katsumatae*, costs were calculated based on human activities. We set the cost value for each planning unit as the anthropogenic disturbance index corresponding to a regular hexagonal planning unit with an area of approximately 1 km^2^ within the study area [29,48]. The level of anthropogenic disturbance is evaluated using a 7-point graded scheme, which is based on remote sensing data and land classification systems and has been extensively validated and refined by numerous experts and scholars [49,50]. The calculation formula is as follows:

(3) $$M=\sum_{i=1}^{n}f_{n}\cdot h$$

In the above equation, M enotes the disturbance index, n represents the anthropogenic disturbance level, h indicates the intensity of anthropogenic disturbance, and $f_{n}$ is the proportion of the area corresponding to the land-use type h at the specified level of anthropogenic disturbance.

We used the Zonae Cogito software to obtain the appropriate SPF and BLM values. In this study, the SPF value was 16.74 and the BLM value was 2.11 [51,52,53]. Given the complexity of defining and estimating the true cost threshold, the CostThresholdPenalty was not considered. We configured the model parameters such that each run generated 1000 solutions. After 1000 iterations of calibration experiments, regions with irreplaceability values exceeding 900 were designated as key protection areas. During the decision-making process of the Marxan model, a conservation target must be set, defined as the minimum proportion of grid cells within the study area that must ultimately be protected. Based on current related research [29,54,55] and taking into full account the unique and endangered status of *P. katsumatae*, this study set a conservation target of 50% for its habitat.

## 3. Results

### 3.1. Evaluating Predictive Accuracy and Environmental Factors Impacts in ESDM

The prediction accuracy of habitat suitability for *P. katsumatae* and three other species, as evaluated by the ESDM, is presented in Table 4. In addition to AUC and TSS, we also listed three other key parameters (Omission.rate, prop.correct, kappa) of the model. The AUC values from the ESDM assessment for all four species exceed 0.8, with the highest value observed for *G. maesi* at 0.881.The TSS values range from a minimum of 0.488 to a maximum of 0.575. Considering that TSS values may be influenced by the accuracy of randomly generated absence data, we deem the model’s prediction results to be highly reliable.

**Figure 2:**
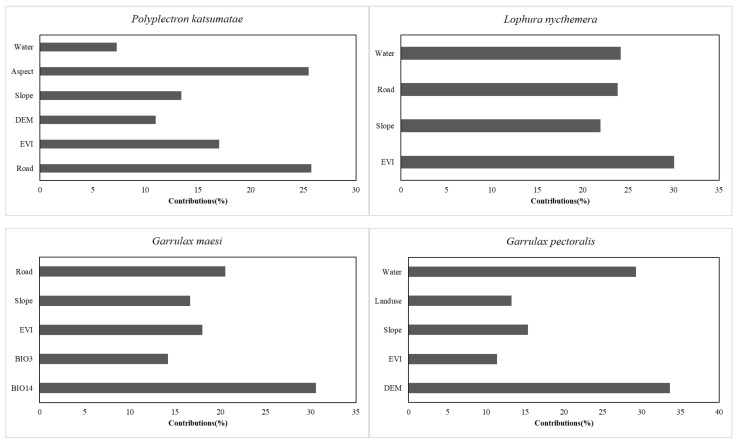
Contributions of environmental factors to the results of habitat suitability prediction for 4 species.

As depicted in Figure 2, environmental factors differentially contribute to the evaluation results across the four species. After screening, a total of nine environmental factors were included in the model evaluation: land use/cover (Landuse), distance from water sources (Water), distance from roads (Road), EVI, elevation (DEM), slope, aspect, isothermality (BIO3), and precipitation of the driest month (BIO14). Among these, BIO3 and BIO14 represent the third and fourteenth parameters, respectively, among the 19 bioclimatic variables. BIO3 is derived from BIO2 (Mean Diurnal Temperature Range) and BIO7 (Temperature Annual Range) using the formula: (BIO2/BIO7 × 100).

For *P. katsumatae*, Road and Aspect are the two most critical environmental factors influencing its habitat selection, each with a contribution rate exceeding 25%, while EVI contributes 17%. Slope, DEM, and Water also exert an influence on the habitat selection of *P. katsumatae*, although their impact is relatively lower. For *L. nycthemera*, four primary environmental factors affect its habitat selection, and the contribution rates of these factors are relatively similar, each exceeding 20%. Among these, EVI has the highest contribution at approximately 30%, followed by Water and Road, both contributing around 24%, while Slope has the lowest contribution, at about 22%. For *G. maesi*, the most important environmental factor affecting its habitat selection is BIO14, which has a contribution rate of 31%. Among the remaining environmental factors, Road exhibits a relatively high contribution of 20%, whereas the contributions of EVI, Slope, and BIO3 range between 14% and 18%. For *G. pectoralis*, DEM and Water are the most critical environmental factors influencing its habitat selection, with contribution rates of approximately 34% and 29%, respectively, while the next most influential factors—Slope, Landuse, and EVI—contribute between 10% and 15%.

**Figure 3:**
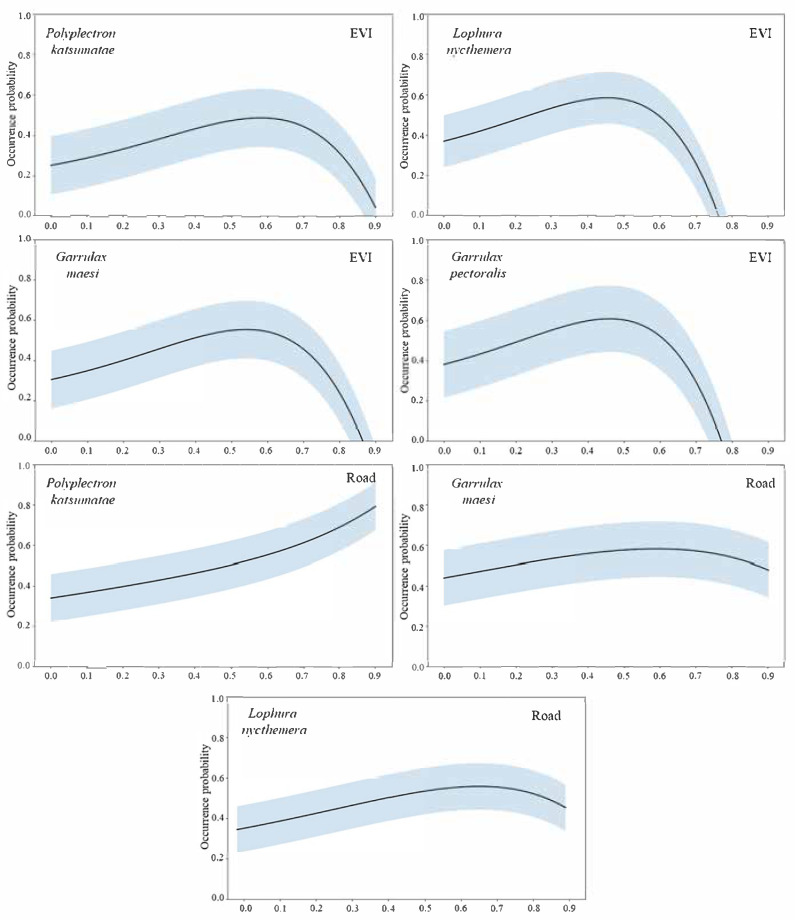
Response curves of typical environmental factors.

Based on environmental variables and habitat suitability assessment results for four species, we generated response curves for all high-contribution environmental factors. Two representative factors (EVI and Road) were selected for detailed visualization, as shown in Figure 3. The *x*-axis represents normalized values (0–1 range) of environmental factors, while the *y*-axis indicates species occurrence probability. Figure 3 (row 1 and row 2) reveals that all four species exhibit a preference for intermediate-to-high EVI values, with both excessively low and high vegetation coverage proving suboptimal. Notably, *P. katsumatae* demonstrates the highest probability peak at elevated EVI levels, suggesting stricter vegetation requirements compared to the other three species. Regarding road proximity, three species display significant avoidance behavior, with occurrence probability declining near roads (Figure 3, row 3 and row 4). *P. katsumatae* exhibits the most pronounced avoidance tendency.

### 3.2. Habitat Suitability Spatial Distribution and Geographic Overlap Analysis

**Figure 4:**
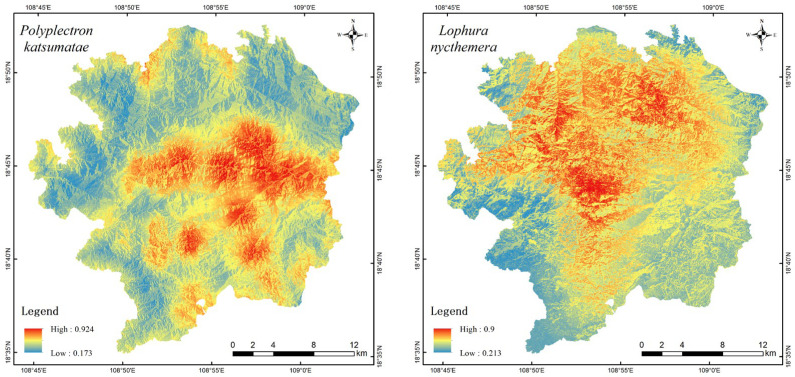

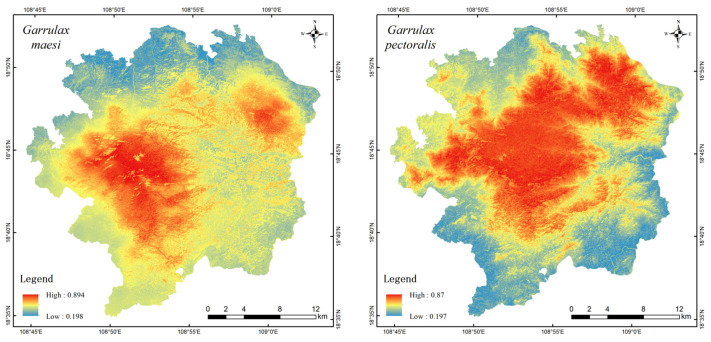
Spatial distribution of habitat suitability for 4 species evaluated by ESDM.

**Figure 5:**
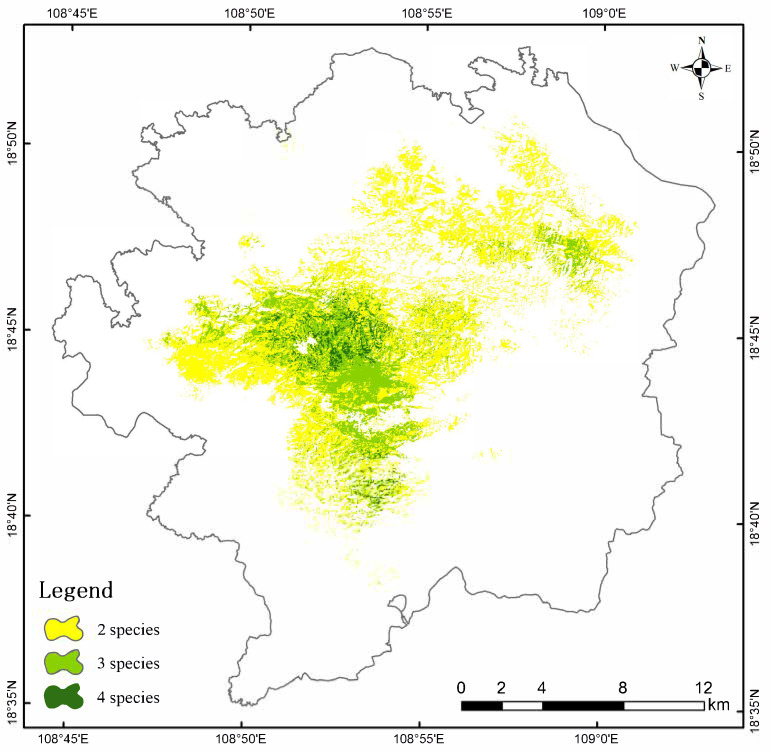
Spatial overlap of suitable habitats among 4 species.

Figure 4 displays the habitat suitability evaluation results for four species obtained through ESDM modeling, where warmer colors (progressing from blue to red) represent higher suitability values. The suitable habitat for *P. katsumatae* is generally concentrated but exhibits a pattern of small, fragmented patches within it. Although suitable habitats are primarily distributed in the central, eastern, and southern parts of the study area, the figure clearly shows that the most optimal habitats occur as small patches. The suitable habitats in the central and eastern regions are well-connected, whereas those in the southern part are relatively isolated with poorer connectivity. The suitable habitat for *L. nycthemera* is mainly distributed in the central and northern regions of the study area. The habitat suitability for *G. maesi* is divided into two distinct areas: a larger patch located in the western and southern parts of the study area, and a smaller patch in the northeastern part. These two areas are relatively isolated from each other. The suitable habitat for *G. pectoralis* has the largest area and is widely distributed across the central, western, northern, and northeastern parts of the study area, forming a relatively large contiguous patch. The spatial distribution of habitat suitability for four species reveals that areas lacking camera trap deployment still demonstrate high habitat suitability, particularly in the peripheral zones of the study area. This observation highlights the capacity of ESDM to partially compensate for spatial sampling biases stemming from non-uniform camera placement strategies. Overall, the distribution of suitable habitats varies considerably among the four species; however, in a small area in the western-central part of the study region, the suitable habitats of all four species overlap, indicating a high-quality habitat at that location. As illustrated in Figure 5, yellow zones represent areas suitable for two species. Light green zones indicate regions accommodating three species, while dark green zones demonstrate habitats concurrently suitable for all four species. Suitable habitats were delineated using a threshold value of 0.7, with areas exceeding this criterion in ESDM assessments classified as habitat-suitable zones for the target species. Collectively, light green and dark green zones predominantly cluster in the central-western region.

### 3.3. Key Protection Areas of P. katsumatae

**Figure 6:**
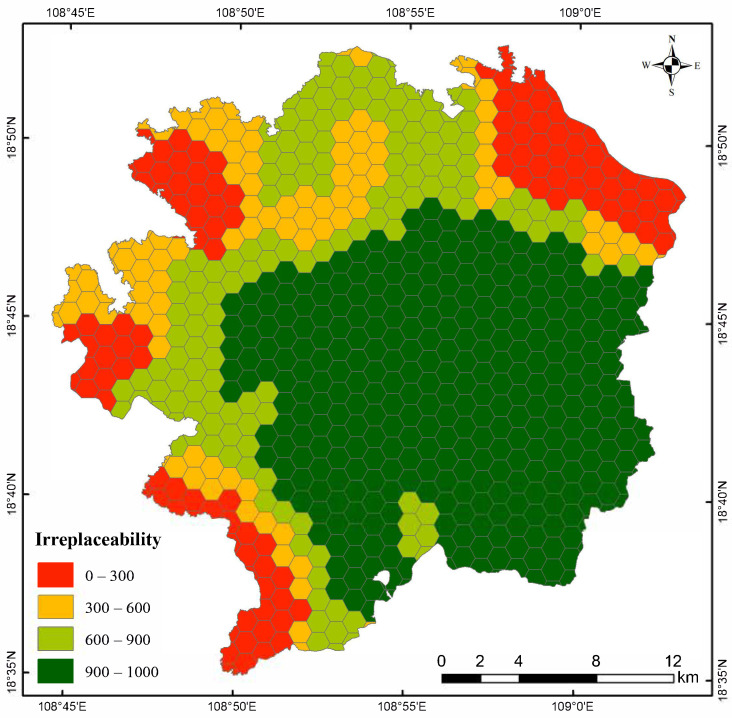
Distribution of irreplaceability values identified by the Marxan model.

**Figure 7:**
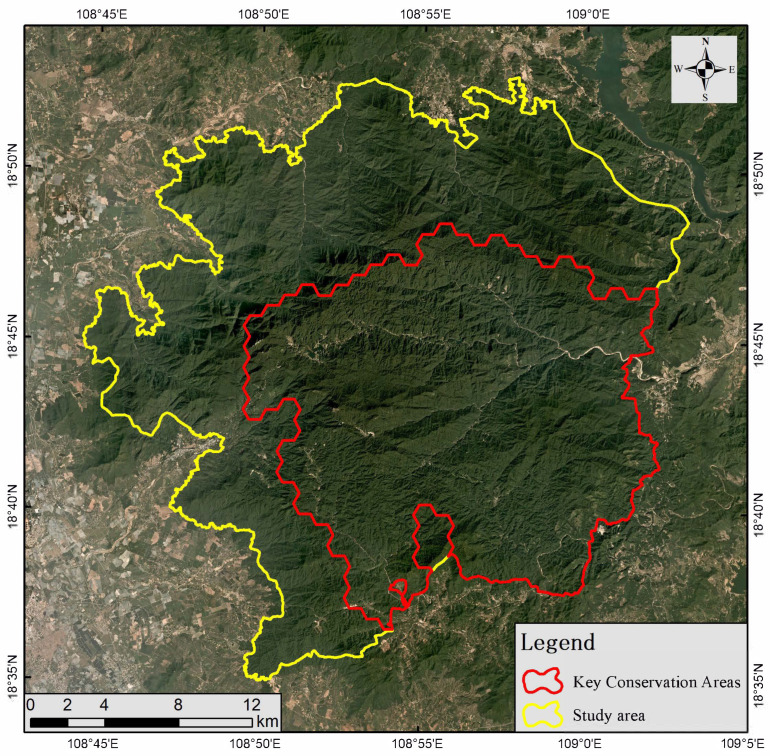
Distribution of key protection areas for *Polyplectron katsumatae* within Jianfengling.

Using the Marxan model and based on the habitat suitability assessment result for *P. katsumatae* obtained from ESDM, the key protection areas for this species were identified. The entire study area was divided into 715 hexagonal planning units (complete or partial). As shown in the irreplaceability value results (Figure 6), regions with high irreplaceability values were primarily concentrated in the central, eastern, and southern parts of the reserve, closely aligning with the distribution of suitable habitats. In this study, key protection areas were delineated based on the top 10% of irreplaceability values, specifically those exceeding 900. The final identified key protection area covered a total of 322.8 km^2^, accounting for 48.9% of the study area (Figure 7).

## 4. Discussion

Analyzing the contribution of environmental factors to habitat suitability for the four ground-dwelling bird species, we found that EVI and Slope influenced all four species. Road and Water were the next most influential factors, affecting the habitat selection of three species. DEM influenced the habitat selection of *P. katsumatae* and *G. pectoralis*. Additionally, four environmental factors—Landuse, Aspect, BIO3, and BIO14—each influenced the habitat selection of a single species. Five environmental factors appeared more than twice, with the average contribution exceeding 15%. Road had the highest contribution at 23.37%, followed by DEM at 22.33%. Considering the frequency of occurrence and average contribution of environmental factors, the habitat selection of these four ground-dwelling bird species was influenced by multiple factors. However, several environmental factors were particularly significant. The first major factor is anthropogenic activity, represented in this study by the Road factor, which indicates the distance from major roads. In reality, human activities in the Jianfengling area primarily fall into two categories. The first is tourism [56], as the main tourist attractions in the study area are accessible by roads, making road presence a proxy for tourism-related disturbances. The second is resource collection and hunting by residents of surrounding villages [14]. Since local villagers are highly familiar with the study area’s environment, they typically use small trails that are not captured in existing road datasets. However, villagers rarely venture deep into the mountains. As a result, human disturbance generally decreases with the increase in the distance from villages and in areas with more rugged terrain. Overall, *P. katsumatae* exhibited the highest sensitivity to human disturbance, with its suitable habitat located the farthest from roads and villages at the periphery of the study area. *L. nycthemera* was also relatively sensitive to human activities. In contrast, *G. maesi* and *G. pectoralis* are relatively smaller in size and more cryptic in their movements, making them less sensitive to human activities compared to the two larger ground-dwelling bird species. Additionally, topographic factors significantly influenced habitat selection. All four species preferred flat areas with low slopes, a pattern consistent with previous studies on ground-dwelling birds [57,58]. Flat areas in tropical rainforests offer greater food availability and better concealment for birds, facilitating their activities. Vegetation factors also play a crucial role in habitat selection [59]. The EVI values in areas where *P. katsumatae* was active were higher than those for the other three species, indicating its preference for densely vegetated forested areas. The suitable habitat EVI values for *G. maesi* and *G. pectoralis* exhibited a wide distribution range, suggesting these species have a broader tolerance for different tropical rainforest types and quality levels. All four species avoided areas with low EVI values, indicating a general preference against inhabiting sparse forests or open areas [60].

In addition to the aforementioned factors, *P. katsumatae* is the only one among the four species that exhibits a pronounced preference for slope aspect in its habitat selection. According to the habitat suitability assessment results, within the Jianfengling study area, *P. katsumatae* tends to favor habitats on east-facing, southeast-facing, and south-facing slopes. Using a GIS-based approach, we spatially integrated suitable habitats with Aspect, EVI, Slope, and DEM to analyze the combined effects of environmental factors on habitat suitability. Typically, sun-exposed slopes—such as south-facing and southeast-facing slopes—in the Jianfengling area tend to have higher temperatures, lower humidity, and stronger weathering, resulting in soils that are relatively poorer than those on shaded slopes. Consequently, the biodiversity of plant communities is generally higher on shaded slopes [14]. However, the joint analysis of Aspect and EVI revealed that suitable habitats for *P. katsumatae* on sun-exposed slopes paradoxically exhibit higher EVI values. The further integration with Slope and DEM showed that these suitable habitats predominantly occur in low-gradient areas at middle elevations. This phenomenon suggests that *P. katsumatae* in Jianfengling has stringent habitat requirements, favoring low, flat, sun-exposed slopes with dense vegetation. Furthermore, synthesizing Aspect, Slope, DEM, and high-resolution remote sensing imagery, we observed that *P. katsumatae* distribution is generally confined to relatively low mountains and valleys, where a limited area is sufficient for the species to traverse regions with varying slopes and aspects. This topographic variation results in a greater vegetation diversity [61], thereby enriching the availability of food sources such as insects and fruits in the area, which in turn provides *P. katsumatae* with a favorable foraging environment. In addition, Considering the diurnal activity rhythm of *P. katsumatae* in Jianfengling, which exhibits a primary activity peak in the morning and a secondary, smaller peak in the evening [14], east- and south-facing slopes provide more favorable light conditions and suitable temperatures in the morning, which is also beneficial for the species’ activity. However, due to its stringent habitat requirements, *P. katsumatae* has fewer distribution points compared to the other three ground-dwelling bird species, resulting in a smaller and more fragmented suitable habitat.

A comparison of the habitat suitability distribution maps for the four species clearly reveals that the suitable habitats for *P. katsumatae* and *L. nycthemera* are relatively independent; one is primarily distributed in the southeastern part of the study area, while the other is mainly found in the northern part. The suitable habitats of *G. maesi* and *G. pectoralis* exhibit some overlap with the suitable habitat of *L. nycthemera*, although the degree of overlap is low. In contrast, these two species show a high level of habitat overlap with each other, indicating significant interspecific competition. Overall, the habitat selection among the four species tends to minimize interspecific competition. Notably, *P. katsumatae*’s suitable habitat shows virtually no overlap with those of the other three species, except for a small area in the central part of the study region where all four species are present and which exhibits the highest habitat suitability and quality. Furthermore, this area is situated far from human settlements and is one of the regions with the highest biodiversity within the study area. However, the current analytical results cannot determine whether the spatial distribution pattern of suitable habitats for *P. katsumatae* is driven by the species’ active habitat selection or results from passive competitive exclusion with other dominant species; further research is necessary to clarify the relationships among these ground-dwelling bird species.

In identifying the key protection areas for *P. katsumatae*, this study fully considered the characteristic dispersion of suitable habitat patches for this species. To maintain the integrity of the key protection areas and ensure connectivity among populations, the Marxan model was configured to recognize only contiguous habitat patches as key protection areas. Ultimately, the identified key protection areas were located in the eastern and southern parts of the research area. This region inherently supports a high density of *P. katsumatae* populations and exhibits a robust ecological baseline, which facilitates effective conservation and restoration efforts for both the species and its habitat. Although the eastern and southern peripheries of this area form part of the boundary of the Hainan Tropical Rainforest National Park (Figure 6), the outer boundary still contains a substantial area of forest vegetation. This serves as an effective ecological buffer, reducing the direct impact of external human activities on the survival of *P. katsumatae*. Moreover, the distribution of other ground-dwelling bird species that compete with *P. katsumatae* in this region is relatively sparse, thereby reducing competitive pressure during population recovery and expansion. Based on these findings, we recommend designating the identified key protection areas as the recovery zones for *P. katsumatae*. We further suggest intensifying conservation and restoration efforts focused on the tropical rainforest ecosystem within these zones, implementing regular monitoring, surveys, and assessments to continuously track the ecological status, increasing patrol efforts, and strictly controlling habitat destruction, illegal planting, collection, and poaching. These measures will provide maximal support for the conservation of *P. katsumatae* and its habitat.

## 5. Conclusions

This study identified fragmented suitable habitats for *P. katsumatae* in the central, eastern, and southern Jianfengling areas of Hainan Tropical Rainforest National Park, primarily attributed to anthropogenic pressures and the species’ specialized habitat requirements. Moreover, the analytical results revealed minimal overlap between *P. katsumatae* habitat and those of *L. nycthemera*, *G. maesi*, and *G. pectoralis*, but the ecological drivers underlying this spatial segregation remain unclear and require further investigation. To address habitat fragmentation and promote population recovery, we propose prioritizing a 322 km^2^ eastern–southern conservation zone, accompanied by enhanced ecological monitoring and stricter regulation of human disturbances.

## Figures and Tables

**Table 1:** Conservation status of the 4 studied species. Roman numerals I/II denote their protection level under national laws or CITES. IUCN threat categories are abbreviated: EN (Endangered) and LC (Least Concern).

Species	No. of Independent Photographs	RAI	National Protected Level	China’s Vertebrate Red List Status	CITES Appendix
*Polyplectron katsumatae*	136	0.320	I	II	EN
*Lophura nycthemera*	723	1.699	II		LC
*Garrulax maesi*	142	0.334			LC
*Garrulax pectoralis*	56	0.132			LC

**Table 2:** Biological characteristics of the 4 species.

Species	Habitat	Diet	Behavior
*Polyplectron katsumatae*	Inhabits tropical rainforests, dense understory [10].	Omnivorous Insects, seeds, fruits [11].	Usually solitary or found in pairs, with a secretive nature adept at avoiding predators [10].
*Lophura nycthemera*	Found in montane forests, bamboo groves, and secondary forests [32].	Omnivorous Seeds, fruits, insects, small invertebrates [32].	Social bird, often seen in pairs or small groups, with strong territorial behavior [33].
*Garrulax maesi*	Resides in lowland evergreen forests, secondary forests, and dense understory [34].	Omnivorous Insects, fruits.	Social bird, typical understory bird, adept at hiding and often seen in groups [34].
*Garrulax pectoralis*	Occupies lowland forests, shrub lands, and bamboo groves.	Omnivorous Insects, fruits, seeds.	Social bird, often seen in noisy groups.

**Table 3:** Datasets used in this study.

Data Types	Specific Data	Data Set	Spatial/Temporal Resolution	Period
Climate data	19 Bioclimatic variables (BIO1~BIO19)	WorldClim Version 2.1	30s (~1 km), month	Average value
Water data	Distance from surface water bodies such as rivers and lakes (Water)	Provided by the protected area management authority (field surveys, remote sensing)	Vector	2019
Road data	Distance from road (Road)	OpenStreetMap (OSM)	Vector	2019
Land use/cover data	Landuse	National Center for Geographic Resources Science	30 m, year	2019
Vegetation data	Enhanced Vegetation Index, (EVI)	LANDSAT/LC08/C01/T1_RT_TOA	30 m, year	2013–2019
Topographic data	DEM/Slope/Aspect	ASETER GDEM v3	30 m	2019

**Table 4:** ESDM model prediction accuracy assessment results.

Species	AUC	TSS	Omission. Rate	Prop. Correct	Kappa
*Polyplectron katsumatae*	0.863	0.565	0.233	0.767	0.511
*Lophura nycthemera*	0.850	0.537	0.247	0.753	0.532
*Garrulax maesi*	0.881	0.488	0.279	0.721	0.446
*Garrulax pectoralis*	0.856	0.575	0.232	0.768	0.530

## Data Availability

The datasets presented in this article are not readily available because the data are part of an ongoing study. Requests to access the datasets should be directed to yaowt@aircas.ac.cn.
